# Identification of Degenerate Nuclei and Development of a SCAR Marker for *Flammulina velutipes*

**DOI:** 10.1371/journal.pone.0107207

**Published:** 2014-09-15

**Authors:** Sun Young Kim, Kyung-Hee Kim, Chak Han Im, Asjad Ali, Chang Yun Lee, Won-Sik Kong, Jae-San Ryu

**Affiliations:** 1 Environment-friendly Research Division, Gyeongsangnam-do Agricultural Research and Extension Services, Jinju, Republic of Korea; 2 Greenpeace Mushroom Co., Chungdo, Republic of Korea; 3 Mushroom Research Division, National Institute of Horticultural and Herbal Science, Rural Development Administration, Eumsung, Republic of Korea; Woosuk University, Republic of Korea

## Abstract

*Flammulina velutipes* is one of the major edible mushrooms in the world. Recently, abnormalities that have a negative impact on crop production have been reported in this mushroom. These symptoms include slow vegetative growth, a compact mycelial mat, and few or even no fruiting bodies. The morphologies and fruiting capabilities of monokaryons of wild-type and degenerate strains that arose through arthrospore formation were investigated through test crossing. Only one monokaryotic group of the degenerate strains and its hybrid strains showed abnormal phenotypes. Because the monokaryotic arthrospore has the same nucleus as the parent strain, these results indicated that only one aberrant nucleus of the two nuclei in the degenerate strain was responsible for the degeneracy. A sequence-characterized amplified region marker that is linked to the degenerate monokaryon was identified based on a polymorphic sequence that was generated using random primers. Comparative analyses revealed the presence of a degenerate-specific genomic region in a telomere, which arose via the transfer of a genomic fragment harboring a putative helicase gene. Our findings have narrowed down the potential molecular targets responsible for this phenotype for future studies and have provided a marker for the detection of degenerate strains.

## Introduction

The basidiomycete *Flammulina velutipes* is one of the most cultivated edible mushrooms. One of the reasons for the recent increase in mushroom production is consumer interest in their high content of macromolecules that possess antitumor, immunomodulatory, and antiviral properties, such as polysaccharides and glycoproteins [1], [2].

Filamentous fungi that are grown on a nutritionally rich medium frequently exhibit instability manifested in morphological and physiological variations. The morphological variations include a lack of or reduction in sporulation, fluffy mycelial-type growths, and variations in hyphal pigmentation [3], [4]. Previous studies implicated infections with double-stranded RNA viruses [5] and the instability of fungal nuclear and mitochondrial genomes in changes in fungal morphology and physiology. Such changes might be expected because fungal genomes are dynamic and capable of a rapid accumulation of genome rearrangements, particularly amplifications and deletions [6]. Likewise, edible mushrooms that were preserved by serial passaging in culture on nutritionally rich media exhibited abnormal mycelial growth and poor yields [7]. Abnormalities in *Agaricus bisporus* led to a lower yield and inferior product quality, resulting in economic losses [8], [9], [10]. These abnormalities may be due to changes in the ribosomal-DNA copy number, the loss of heterozygosity at specific loci, de-heterokaryotization, chromosomal loss, or chromosomal-length polymorphisms [10]. In the case of *F. velutipes*, abnormalities such as malformed fruiting bodies and the complete loss of fruiting-body development have been observed [11]. Despite the relative frequency of these occurrences, the underlying cause is not entirely clear. Notwithstanding its economic importance, many basic questions involving the biology of this fungus remain unanswered; hence, a method for detecting mutant strains of *F. velutipes* would be very useful. *F. velutipes*, unlike many basidiomycetes, produces abundant arthrospores in both monokaryotic and dikaryotic mycelia [12]. Moreover, most of the arthrospores contain only one haploid nucleus, which is generated without resorting to fruiting-body formation and chromosomal rearrangements [13]. Thus, the normality of each nucleus can be easily determined by test crossing. Additionally, molecular selection markers such as sequence-characterized amplified regions (SCARs) are required to assist mushroom farmers in cultivating wild-type strains rather than degenerate clones. Herein, we describe the characterization and identification of a degenerate nucleus and report a specific marker for differentiating wild-type and degenerate nuclei.

## Materials and Methods

### Strains and pure cultures

The wild-type (Fv1-5) strain and 3 degenerate strains (Fv1-5^d1^, Fv1-5^d2^, and Fv1-5^d3^) of *F. velutipes* were obtained from various mushroom farms (Table 1). The mycelia were grown on mushroom complete medium (MCM) at 25°C. Because *F. velutipes* mycelia are typically transferred as a plug rather than a single cell, the collected degenerate strains most likely contained some normal cells. To enhance the purity of the degenerate strains, the mycelia were serially cultured on modified BTB-sawdust agar containing 0.45% peptone, 0.75% yeast extract, 0.5% oak sawdust, 0.025% bromothymol blue (BTB, Sigma-Aldrich, St. Louis, MO, USA), and 2% agar, growth on which has been reported as an indicator of normal function [11]. At each round of isolation, the mutants that did not decolorize the BTB agar and exhibited abnormal morphologies, such as a reduced growth rate and compact mycelial colonies, were transferred to fresh medium via cells at the end of the mycelia. The isolated degenerate and wild-type strains were grown on MCM agar for subsequent analyses, and their mycelial growth rates along a perpendicular line were determined at 3, 5, and 7 d (dikaryons) or at 8, 10, and 15 d (monokaryons) after inoculation. The experiments were performed in triplicate.

**Table 1:** List of the *F. velutipes* strains used in this study.

Strain	Description	Stock No.*
Fv1-5	Wild-type, cultivar	KCCM 90233
Fv1-5^d1^	Degenerate dikaryon	KCCM 90234
Fv1-5^d2^	Degenerate dikaryon	-
Fv1-5^d3^	Degenerate dikaryon	-
W1×W4	Hybrid between monokaryons from a wild-type strain	KCCM 90239
W1×D4	Hybrid between monokaryons from wild-type and degenerate strains	KCCM 90242
D1×W4	Hybrid between monokaryons from wild-type and degenerate strains	KCCM 90240
D1×D4	Hybrid between monokaryons from a degenerate strain	KCCM 90241
W1	Monokaryon isolated from Fv1-5, *AxBy*	KCCM 90235
W4	Monokaryon isolated from Fv1-5, *AyBx*	KCCM 90236
D1	Monokaryon isolated from Fv1-5^d1^, *AxBy*	KCCM 90237
D4	Monokaryon isolated from Fv1-5^d1^, *AyBx*	KCCM 90238

*: KCCM: Korean Culture Collection of Microorganisms.

### Monokaryon isolation, test crossing, and fructification

The purified wild-type and degenerate strains were grown on MCM agar for 7 d at 25°C. Arthrospores were obtained from the solid medium by adding 1 to 2 mL of sterile water to the cultures and gently prodding them for a few seconds. The suspension was serially diluted, plated onto MCM agar after filtering through glass wool, and grown at 25°C. The germinated mycelia were isolated, and the clamp connections were observed using phase-contrast microscopy at 40× magnification (Olympus IX71; Olympus, Tokyo, Japan). Monokaryons with no clamp connection were subjected to reciprocal crossing to identify their mating type (Table 1). To determine the abnormal monokaryon group in the two compatible groups that were derived from the degenerate strain, all possible test-crossing combinations were performed between monokaryons derived from the wild-type and degenerate strains. The hybrids were subjected to growth-rate analysis and the fruiting test. A substrate containing pine sawdust (23%), corncob (29%), rice bran (18%), beet pulp (4%), wheat bran (14%), cottonseed hull (4%), shell powder (4%), and soybean powder (4%), with a 65% water content, was autoclaved at 121°C for 1.5 h, cooled to 20°C, inoculated with four plugs (1×1 cm) of cultured hybrids, and finally placed in an incubation room maintained at 19°C to 20°C for spawn running. On the 35th day of the spawn running period, the outer area of the substrate was removed by scraping to induce the formation of primordia. Then, the cultures were placed in a cultivation room that was maintained at 15°C and 90% relative humidity to induce fruiting. When the fruiting bodies reached a length of approximately 2 cm, the temperature was lowered to 5°C for 5 to 7 d to normalize the size of the fruiting bodies. Subsequently, the cultures were maintained at 10°C with 75 to 80% relative humidity (RH) to allow the fruiting bodies to grow.

### Extraction of genomic DNA

Genomic DNA was extracted from dikaryons and monokaryons using the Exogene Plant SV kit (GeneAll Biotechnology, Korea) according to the manufacturer's protocol. The yields of DNA were quantified using a NanoDropND-1000 UV spectrophotometer (NanoDrop Technologies, Wilmington, DE, USA).

### Identification of an abnormality-associated marker

A draft genomic sequence of the monokaryotic strain (Mono3 derived from meiotic spore of Fv1-5) of *F. velutipes* that was obtained in a previous study (http://112.220.192.2/fve/) and the complete genomic sequence of KACC42780 [14] were used as sources for SSR marker development and comparative analysis. The SSR candidates were identified using SSR Locator I [15]. Primers were designed accordingly, using the Primer3 program [16]. Random-amplified polymorphic DNA (RAPD) PCR was performed in 20-µL mixtures containing 30 ng of genomic DNA, 1× e-Taq buffer, 0.2 mM dNTPs, 0.2 µmol of Taq polymerase (Solgent, Korea), and 1 µM random primers of the OPB, OPC, OPD, OPE, OPF, OPG and OPI series (Operon Tech., California, USA). PCRs were performed using a Gene Atlas system (ASTEC, Japan) and the following protocol: an initial denaturation step of 5 min at 95°C; followed by 40 cycles of 1 min of denaturation at 95°C, 1 min of annealing at 40°C, and a 2-min extension at 72°C; with a final extension step of 5 min at 72°C. The SSR PCR cycles consisted of an initial denaturation step of 3 min at 95°C; followed by 35 cycles of 20 s at 95°C, 40 s at 52°C, and 30 s at 72°C; with a final step of 5 min at 72°C. The PCR products were resolved using 1% (RAPD) and 3% (w/v: SSR) agarose gels (Life Technologies, USA) in TAE buffer (400 mM Tris, 200 mM sodium acetate, and 20 mM EDTA, pH 8.3) containing RedSafe (Intron, Korea). The specific degenerate DNA bands were excised from the gel, purified using an Expin PCR SV kit (GeneAll Biotechnology, Korea) as described in the manufacturer's protocol, ligated into a vector using a Dr. TA TOPO cloning kit (Doctor Protein, Korea) and were then used for bacterial transformation. The recombinant plasmids were isolated using a Plasmid DNA purification Hybrid-Q kit (GeneAll Biotechnology, Korea) and were sequenced (Macrogen Corp, Korea). To increase the reproducibility and reliability of the results obtained using the random primer set, we constructed a SCAR marker (22-mer) based on the sequence of the polymorphic RAPD band (Table S1).

### Comparative analysis

The acquired sequences specific to the degenerate monokaryons were aligned with the genomic sequences of the monokaryotic strain Mono3 using the DNAMAN program (Lynnon Corp, Canada) to determine its flanking sequence. Because the degenerate-specific region of the degenerate (D4) strain was determined according to scaffold 59 of the Mono3 genomic sequence, PCR was performed using primer sets spaced every 1 kb (for the sequence from 1 to 10 kb), 10 kb (for the sequence from 10 to 60 kb) or 60 kb (for the sequence from 60 to 121 kb) based on the scaffold 59 sequence for synteny analysis of the wild-type (W4) and D4 strains. To determine the genomic region corresponding to the degenerate-specific sequence and the boundary regions for the W4 and D4 genomic sequences, the primer sets for 9,244 to 10,004 and 9,500 to 10,500 and other regions were used to amplify DNA fragments, which were sequenced (Table S1). PCR using the same mixture described above was performed in 20 µL volumes using the following protocol: initial denaturation for 5 min at 95°C, followed by 35 cycles of 1 min denaturation at 94°C, 1 min annealing at 60°C, and a 90-s extension at 72°C. The PCR products were evaluated using electrophoresis on 1% (w/v) agarose gels. The PCR products were ligated into a vector using a Dr. TA TOPO cloning kit and sequenced in the forward and reverse directions using M13 primers. The sequences were assembled using the DNAMAN program (Lynnon Corp). Three sequences from the W4, D4 and KACC42780 strains were aligned using the DNAMAN program. The sequence specific to the D4 strain was analyzed to predict the presence of genes and the functions and domains of the gene products using the FGENESH program (http://www.softberry.com/), the UniProt program (http://www.uniprot.org/) and the Protein BLAST program from the NCBI. Tandem repeats in the genomic DNA were identified using the Tandem Repeats Finder program (http://tandem.bu.edu/trf/trf.html).

## Results and Discussion

### Growth characteristics and morphological traits of the degenerate mycelium

**Figure 1:**
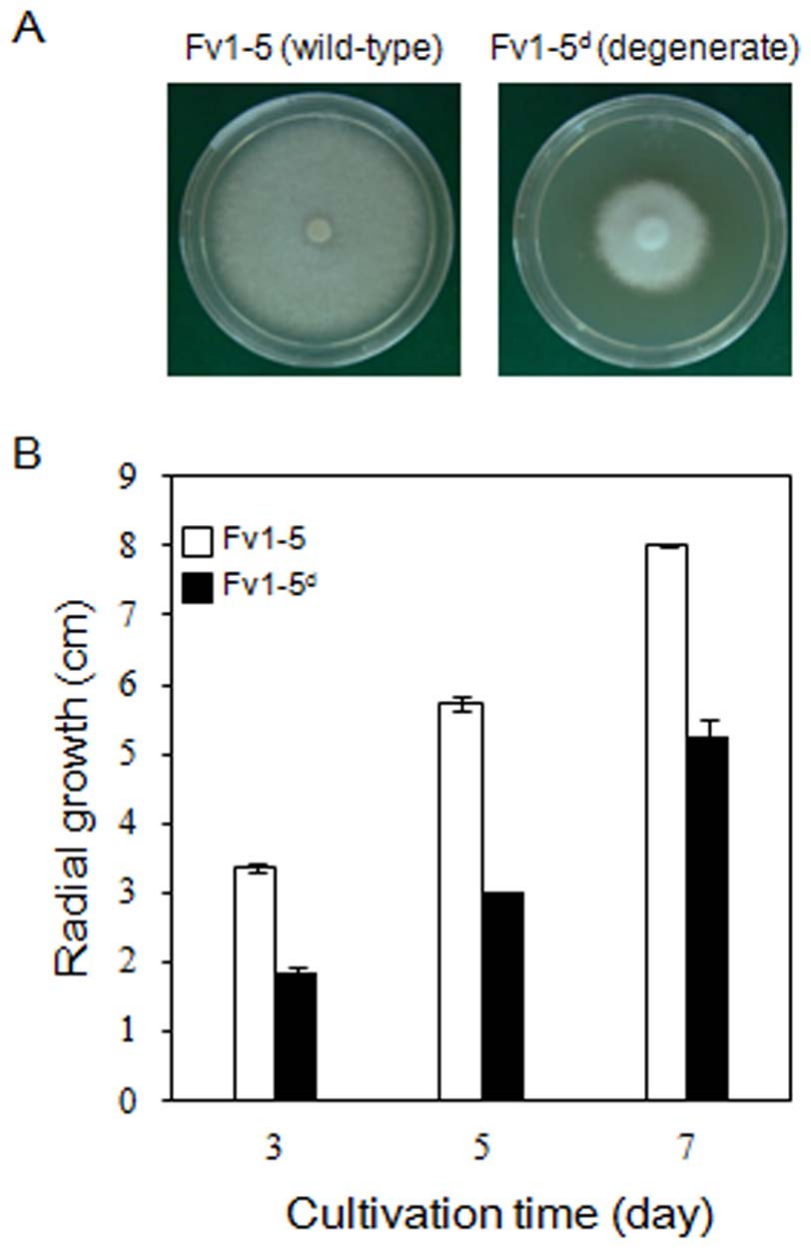
Mycelial growth of wild-type and degenerate dikaryons of *F. velutipes*. (A) Morphology of the mycelia of the wild-type (left) and degenerate strains (right) that were grown on MCM agar for 7 d. (B) Growth rates of the wild-type and degenerate strains were determined based on the diameter of the mycelia grown at 25°C. The error bars represent the mean standard deviations of triplicate samples.

The degenerate strains exhibited abnormal phenotypes, including reduced growth rates and the formation of compact mycelial mats compared to the wild-type strain (Fig. 1A); however, the extent of these abnormalities was strain-dependent (data not shown). The wild-type *F. velutipes* strain elicited a color change in BTB from blue to yellow, whereas the degenerate strains did not [11]. The results of the decolorization of the dye by these fungi suggest that laccase and peroxidase were responsible for the degradation activity via oxidative reactions [17]. The phenolic structure of BTB, which includes a methyl group, is similar to that of lignin and is targeted by ligninolytic enzymes just as lignin is degraded [18]. During the initial transfers (i.e., within the first and second rounds of transfer), the non-decolorized regions were scattered at the edge of the growing mycelial colony in the degenerate strains, whereas the wild-type strain completely decolorized the BTB. The decolorization activity was enhanced when the MCM + BTB was supplemented with sawdust (data not shown). After serial transfers of the non-decolorizing regions to medium supplemented with BTB and sawdust, the non-decolorizing strains Fv1-5^d1^, Fv1-5^d2^, and Fv1-5^d3^, which exhibited consistent phenotypes throughout the mycelial mats, were obtained. The growth rate of the degenerate mycelia (Fv1-5^d1^) was 1.87 cm/3 d, whereas that of the wild-type mycelia was 3.36 cm/3 d (Fig. 1B). Poor quality and yield have also been reported to be accompanied by the abnormal microscopic morphology of the mycelia of *A. bisporus*, including the branching pattern and thickened hyphae [8], [9]. However, no difference between the wild-type and the mutant strains was observed in our study, consistent with the results of the study of Magae et al. (2005) on mycelial morphology at the microscopic level (data not shown).

**Figure 2:**
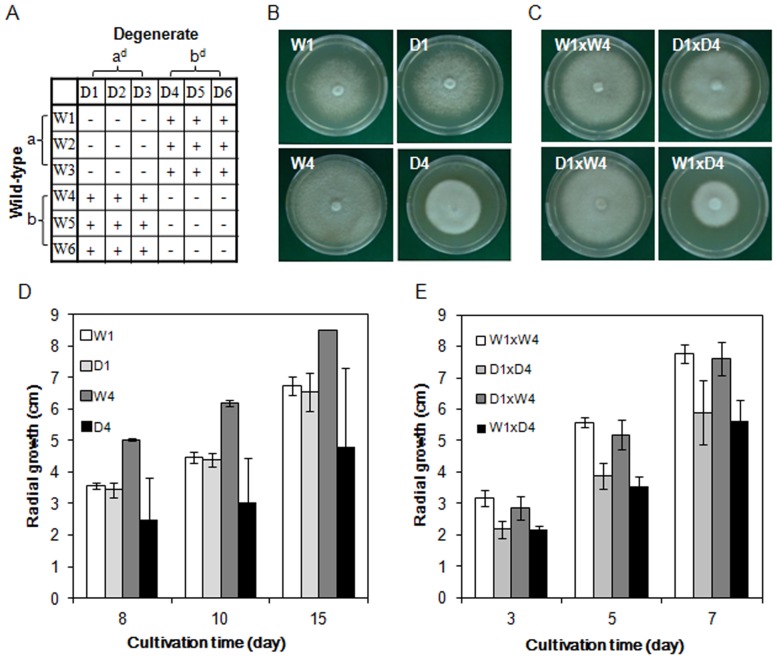
Growth of monokaryotic mycelia and test crosses in *F. velutipes*. (A) Crossing table of the monokaryons derived from the wild-type or degenerate strains. (B) Morphology of the mycelia of the degenerate monokaryons (right) and their wild-type (left) counterparts. W1 and D1 (upper panel) and W4 and D4 (lower panel) have compatible relationships. (C) Mycelial morphology of hybrids from crosses between monokaryons of wild-type and degenerate strains. The growth rates of the monokaryon (D) and hybrid test crosses (E) were determined based on the diameter of mycelia grown at 25°C.

To identify the abnormal-nucleus group, two compatible monokaryons of the wild-type strain and the degenerate strains were prepared through arthrospore formation followed by mating. Three isolates of each mating group of the wild-type and degenerate strains were selected for further study. The mating types were divided into two compatible categories, with the wild-type group “a” (W1, W2, and W3) and the degenerate group “a^d^” (D1, D2, and D3) on one side and the wild-type group “b” (W4, W5, and W6) and the degenerate group “b^d^” (D4, D5 and D6) on the other (Table 1, Fig. 2A). The growth rate of the mycelia in group b^d^ was less than half that of its wild-type counterpart, but the mycelial mat of the former was more compact (Fig. 2B). In contrast, the morphology (Fig. 2B) and growth rate (Fig. 2D) of the other monokaryons (a^d^) were not distinguishable from those of the wild-type group (a). To elucidate the effect of monokaryotic abnormalities on the dikaryon, hybrids were obtained from inter- and intra-compatible combinations of the monokaryons of Fv1-5 and Fv1-5^d1^. Clamp connections were observed despite the presence of chromosomal abnormalities (data not shown), indicating that the formation of clamp connections is not influenced by the degeneration of these strains. The hybrids a×b^d^ (W1×D4, W2×D5, W3×D6) and a^d^×b^d^ (D1×D4, D2×D5, and D3×D6) exhibited phenotypes similar to those of Fv1-5^d1^, including slow propagation of the mycelia and the formation of a mycelial mat on the MCM agar plates (Figs. 2C, 2E). The phenotypes of the hybrids (a×b and a^d^×b; W1×W4, W2×W5, W3×W6, D1×W4, D2×W5, and D3×W6) were not significantly different from those of the wild-type hybrids. Previous reports [19], [20] have identified mitochondrial disorders as causative agents of mycelial abnormalities, but our results excluded that possibility because the group a^d^ and b^d^ monokaryons shared an identical mitochondrial pool, yet only one group showed a distinctly degenerate phenotype.

### Fruiting-body formation by the hybrids

**Figure 3:**
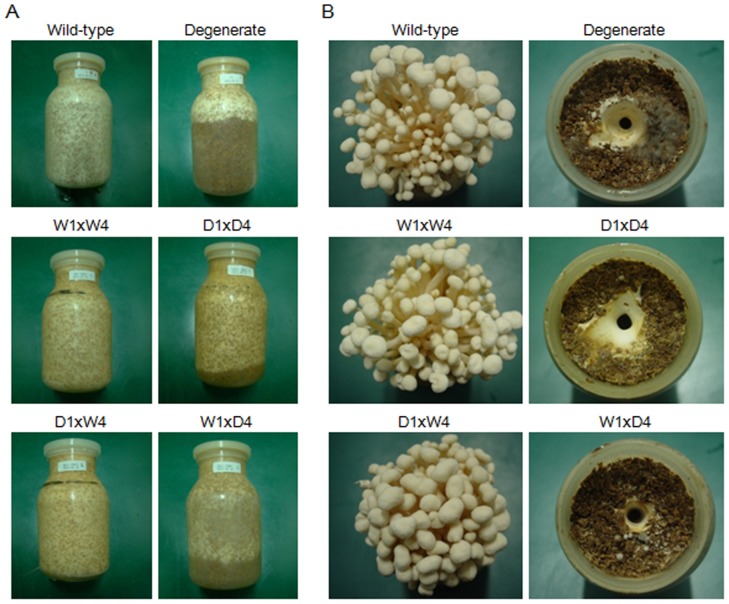
Mycelial running and formation of fruiting bodies. (A) Mycelial running of the wild-type and degenerate dikaryons and the hybrids of crosses between monokaryons of the wild-type (Fv1-5) and degenerate (Fv1-5^d^) strains grown on sawdust medium in bottle cultures for 35 d at 20°C after inoculation. (B) Morphologies of the fruiting bodies produced by the strains represented in the left panel. The experiments were performed using triplicate samples.

To compare the fruiting-body formation of the wild-type and degenerate monokaryons, hybrid mycelia were cultivated in bottles on sawdust medium at a commercial scale. The D1×W4 hybrid showed spawn running and developed characteristics similar to those of the wild-type strain (Fig. 3A). In contrast, for the W1×D4 hybrid, the spawn did not completely run on the sawdust medium at 35 d nor were primordia or fruiting bodies observed (Fig. 3B). Other sibling combinations of W1×D4 showed consistent results (data not shown). In contrast, the hybrids (the a×b group and the a^d^×b group) showed normal spawn running, primordial emergence, pinheading, and fruiting-body development that were undistinguishable from those of the wild-type strain (Fig. 3A, B). Considering the abovementioned observations, the b^d^ group appeared to be responsible for the serious defect in fruiting-body differentiation.

### Identification of an abnormality-associated marker

**Figure 4:**
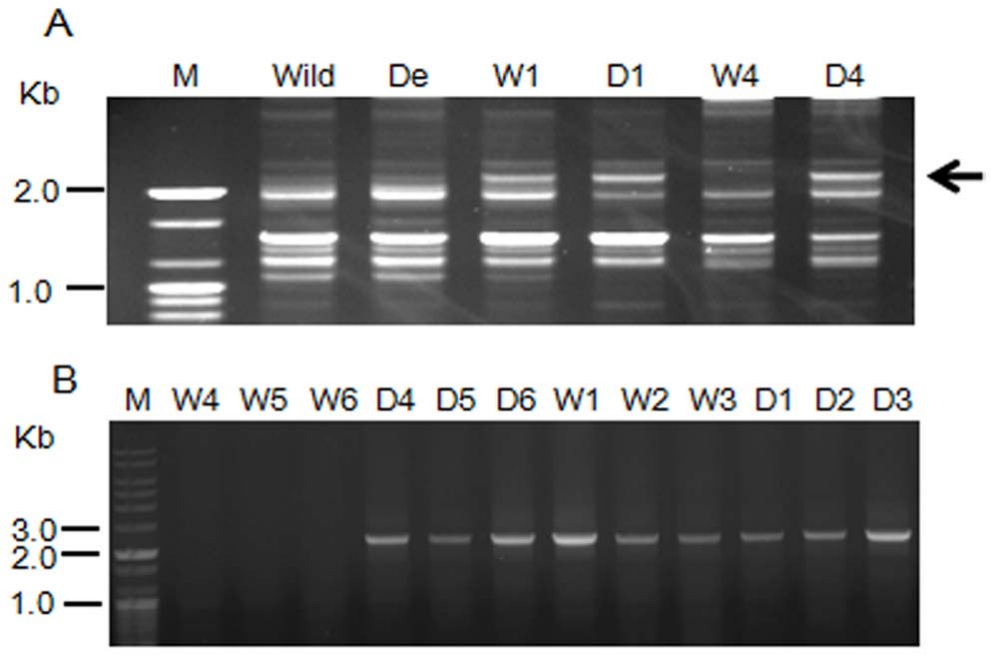
PCR-amplification products of the RAPD and SCAR primers. (A) RAPD electrophoretic profile of monokaryons of the wild-type and degenerate strains obtained using random primer OPD-05. The arrows indicate the polymorphic bands of the wild-type (W4) and degenerate monokaryons (D4). (B) Specificity of the FEDE primer set (SCAR marker). Wild: Fv1-5 (wild-type), De: Fv1-5^d^ (degenerate). W1 to W6 are monokaryons from Fv1-5, and D1 to D6 are monokaryons from Fv1-5^d^. W1-W3: group a (*AxBy*), W4-W6: group b (*AyBx*), D1-D3: group a^d^ (*AxBy*), and D4-D6: group b^d^ (*AyBx*).

Polymorphisms were frequently detected in the RAPD PCR products of compatible monokaryons (groups a^d^ and b^d^), whereas polymorphisms were very rare in the RAPD PCR products of groups b and b^d^. Of the 438 primers tested, only the OPD-05 primer detected a polymorphism at approximately 2.1 kb (Fig. 4A). The SCAR marker was designed based on a specific amplified sequence obtained from a single fragment (Fig. 4B), which appears to be present in the b^d^, a, and a^d^ strains but not in the b strain. However, this marker could not discriminate between the dikaryotic wild-type and dikaryotic degenerate strains. The SCAR-marker primer set consistently amplified the degenerate-specific band from other monokaryons (the same mating group as b) of the degenerate strains (Fv1-5^d2^ and Fv1-5^d3^) (data not shown). These results suggested that the transfer of a genomic region that included the SCAR primer binding sites from an a strain nucleus to a b strain nucleus. Although this screening procedure was not applied to other *F. velutipes* cultivars in the present study, Fv1-5 is a widespread cultivar in Asia, and the degenerate strains collected from farms exhibited similar abnormalities, including compact mycelia and slow growth. Thus, our procedure will be helpful to commercial farmers for distinguishing degenerate mycelia from the complex mixture of normal and degenerate structures that are frequently found on mushroom farms.

### Genomic localization of the region responsible for the abnormalities

**Figure 5:**
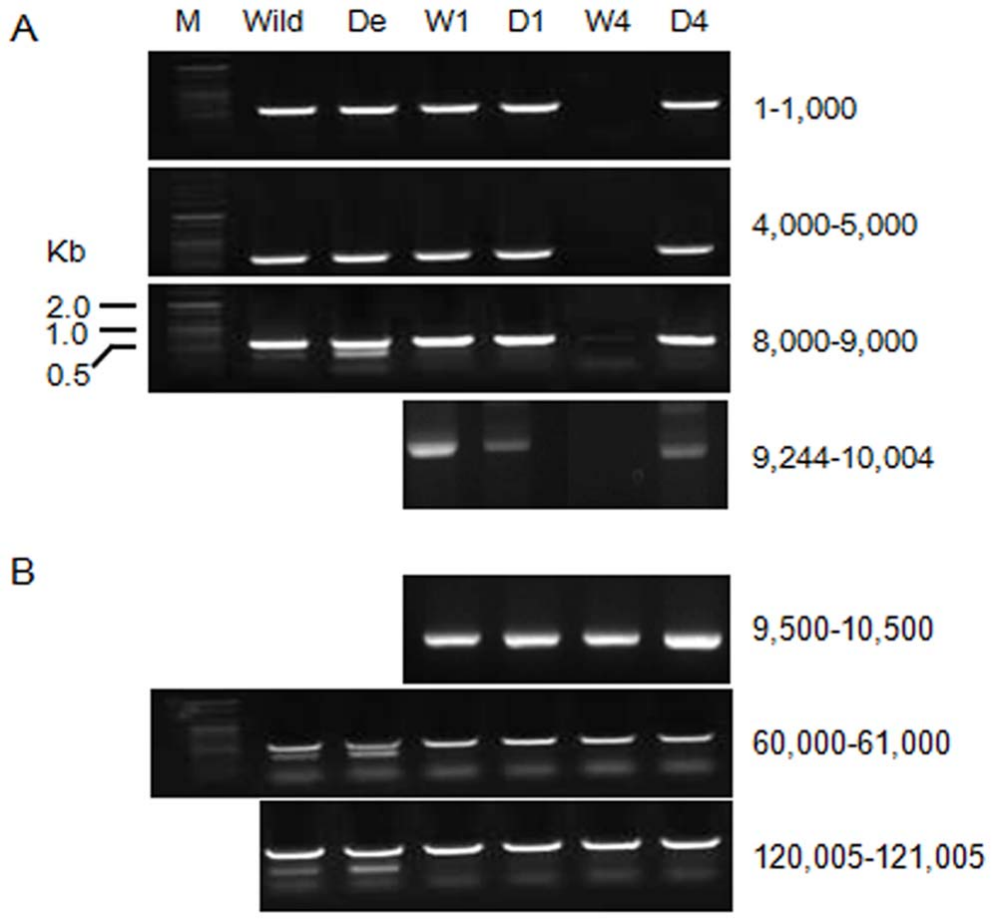
Synteny analysis of the genomic regions of D4 and W1 specific to the degenerate strain. (A) DNA fragment of the W4 and D4 genomic region corresponding to 1 to 10 kb of scaffold 59 from the *F. velutipes* monokaryotic strain (Mono3) obtained by PCR using the SCAR primer set (FEDE-F and -R). Wild: Fv1-5 (wild-type), De: Fv1-5^d^ (degenerate). W1 to W6 are monokaryons from Fv1-5, and D1 to D6 are monokaryons from Fv1-5^d^. W1–W3: group a (*AxBy*), W4–W6: group b (*AyBx*), D1–D3: group a^d^ (*AxBy*), D4–D6: group b^d^ (*AyBx*). (B) DNA fragment of the W4 and D4 genomic region corresponding to 60 to 121 kb of scaffold 59 obtained by PCR using the SCAR primer set.

The alignment of the sequence acquired from D4 with the Mono3 genomic sequence [21] showed more than 99% sequence similarity with bases 2,859 to 5,041 of scaffold 59 (data not shown). Based on the synteny analysis of the wild-type and degenerate genomic regions corresponding to this scaffold, the region from approximately 1 to 10 kb showed that specific bands of the expected size could be detected in D4 but not in its counterpart W4 (Fig. 5A). In contrast, similar band patterns in the wild-type and degenerate strains were detected in the regions beyond 10 kb (Fig. 5B). To determine the boundary region, PCR was performed using the primers that hybridized at 9,244 bp in the genomic sequence that only yielded a band for D4. However, primers that hybridized at 9,500 bp yielded amplicons for D4 and W4, indicating that the boundary region of the wild-type and degenerate genomic region lie between 9,244 and 9,500 bp (Fig. 5A, B). The alignment of the sequences from D4, W4 and KACC42780 showed that the region corresponding to the degenerated region (1 to 9,244 bp of scaffold 59) was detected only in the D4 genome, indicating the involvement of this region in the degenerate phenotypes (Fig. S1, S2). Comparative analysis of the genomic region between 9.5 to 9.7 kb of D4 with that of the KACC42780 genome revealed that the location of the degenerate-specific region is near the terminus of chromosome 8 (−116 kb from the end) (Fig. S2). At the 3' terminus of the D4 genome, multiple telomeric repeating units (TTAGGG) that were found in the telomeric region of *P. ostreatus* (Perez. 2009) were detected (Fig. S1), indicating that the degenerate-specific sequence is likely to be a telomeric region. The similarity between this region in scaffold 59 (Mono3) and its corresponding region in Ch8 (KACC42780) ranged from 30 to 79% because Mono3 is a highly inbred cultivar, whereas KACC42780 was isolated as a wild-type strain.

Based on the FGENESH, UniProt and protein blast analyses, the region corresponding to the degenerate-specific genomic region of the D4 strain encodes a protein of 2,432 amino acids and a molecular weight of 268 kDa, which showed similarity to the putative ATP-dependent helicase C17A2.12 of *Thanatephorus cucumeris* and contains HELICc and HepA domains (Fig. S1). These helicases are reportedly involved in various functions, such as replication, transcription, translation, recombination, DNA repair, and ribosomal biogenesis [22]. Abnormal helicase activity causes several developmental defects in fungi and plants, such as slow growth, premature aging and aberrant assembly of chromosomes [23], [24]. These observations suggest a close relationship between the abnormalities observed and this helicase. The absence of a 1–10 kb sequence from the W4 genome (wild-type) and its presence in the telomeric region in the D4 genome (degenerate), which displays a high rate of recombination and translocation in *P. ostreatus*, yeast, and humans [25], [26], [27], supports the hypothesis that a genomic region harboring a putative helicase was transferred from an a strain nucleus to a b strain nucleus to form the b^d^ strain. Thus, this putative helicase might be required for vegetative growth and fruiting-body development in *F. velutipes*. Likewise, the transfer of the foreign helicase gene might imbalance its activity and result in the observed abnormalities. However we cannot rule out the possibility that other region is responsible for the degenerations because F. velutipes genome is e (35 MB)Chromosomal abnormalities can cause the degeneration of mushrooms [10], [29]. In fungi, extraordinary chromosome rearrangement resulting from insertion/deletion, duplication and translocation within and among chromosomes has been reported (for review, see [28]). Some of these chromosomal alterations may cause fungi to gain a new pathogenicity or adaptation to a novel environment [28]. However, in mushroom fungi, in which strain stability is critical for stable cultivation, genomic rearrangement should be avoided. Recombination predominantly occurs during meiosis, but this strain of edible mushrooms likely degenerated during serial passaging (mitosis). In further studies, the mechanism by which a genomic region from an a group cell was transferred to a b group nucleus to form a degenerated b (b^d^) strain should be addressed. Moreover, *F. velutipes* produces abundant arthrospores in both monokaryotic and dikaryotic mycelia [12] on media used in farms. Thus, the nucleus from a group of mycelia can be transferred to a diploid strain (Fv1-5) via plasmogamy. We attempted to identify the mobile elements that may cause genomic rearrangements [29], [30] throughout the flanking sequences of the putative helicase gene, but no such element was detected. Somatic recombination has been reported in fungi [29] and may be a possible explanation for the mechanism by which a genomic region from an "a" group strain, corresponding to degeneration, is integrated into a "b" strain genome. The results of the present study might be helpful for identifying the potential molecular targets responsible for the degenerate phenotype. Further studies are required to determine whether helicases are directly involved in the abnormalities of *F. velutipes* and to confirm the mechanism by which a genomic region is transferred from one nucleus to another.

## Supporting Information

Figure S1 **Alignment of the sequences corresponding to the degenerate-specific region from the degenerate (D4), wild-type (W1) and KACC42780 strains.** Gene prediction was conducted using FGENESH, and the protein function and domain analyses were conducted using the UniProt (http://www.uniprot.org/) program and the Protein BLAST program of NCBI. The exons of the putative helicase gene are shown in bold and the start and stop codons are underlined. * indicates the residues that are identical in the three sequences. ▾ and arrows indicate the sequence amplified using the SCAR marker primers that discriminated the degenerates. ∇ and arrows indicate the genomic sequences corresponding to the HELICc and HepA domains. The telomeric repeating units (TTAGGG) are in shaded boxes.
(PDF)

Figure S2 **Deduced map of the genomic region corresponding to the degenerate-specific sequence of the wild-type (W1), degenerate (D4) and KACC42780 strains.** Unknown but deduced sequences are shown in boxes indicated with a broken line. The arrow indicates the direction of transcription of the putative helicase gene. The black boxes in the W1 and D4 sequences indicate high similarity (99%), and the gray boxes indicate low similarity (30 to 70%).
(TIF)

Table S1 **List of primers used in this study.**
(DOCX)

## References

[1] Yang J-H, Lin H-C, Mau J-L (2001) Non-volatile taste components of several commercial mushrooms. Food Chem 72: 465–471.

[2] Ko W-C, Liu W-C, Tsang Y-T, Hsieh C-W (2007) Kinetics of winter mushrooms (*Flammulina velutipes*) microstructure and quality changes during thermal processing. J Food Eng 81: 587–598.

[3] Kale S, Bennett J (1992) Strain instability in filamentous fungi. Handbook of applied mycology 5: 311–331.

[4] Wang C, Butt TM, St Leger RJ (2005) Colony sectorization of *Metarhizium anisopliae* is a sign of ageing. Microbiology 151: 3223–3236. 16207906 10.1099/mic.0.28148-0

[5] Dawe AL, Nuss DL (2001) Hypoviruses and chestnut blight: exploiting viruses to understand and modulate fungal pathogenesis. Annu Rev Genet 35: 1–29. 11700275 10.1146/annurev.genet.35.102401.085929

[6] Galagan JE, Henn MR, Ma L-J, Cuomo CA, Birren B (2005) Genomics of the fungal kingdom: insights into eukaryotic biology. Genome Res 15: 1620–1631. 16339359 10.1101/gr.3767105

[7] Singer R (1961) Mushrooms and truffles: botany, cultivation, and utilization. L. Hill, London.

[8] Li A, Begin M, Kokurewicz K, Bowden C, Horgen PA (1994) Inheritance of strain instability (sectoring) in the commercial button mushroom, *Agaricus bisporus* . Appl Environ Microbiol 60: 2384–2388. 16349322 10.1128/aem.60.7.2384-2388.1994 PMC201660

[9] Heath MC, Li A, Horgen PA, Tam PL (1995) Hyphal morphology associated with strain instability in the commercial mushroom, *Agaricus bisporus* . Mycologia 87: 442–450.

[10] Horgen PA, Carvalho D, Sonnenberg A, Li A, Van Griensven LJAD (1996) Chromosomal anormalities associated with strain degeneration in the cultivated mushroom, *Agaricus bisporus* . Fungal Genet Biol 20: 229–241.

[11] Magae Y, Akahane K, Nakamura K, Tsunoda S (2005) Simple colorimetric method for detecting degenerate strains of the cultivated basidiomycete *Flammulina velutipes* (Enokitake). Appl Environ Microbiol 71: 6388–6389. 16204563 10.1128/AEM.71.10.6388-6389.2005 PMC1266012

[12] Ingold C (1980) Mycelium, oidia and sporophore initials in *Flammulina velutipes* . Trans Br Mycol Soc 75: 107–116.

[13] Kemp R (1980) Production of oidia by dikaryons of *Flammulina velutipes* . Trans Br Mycol Soc 74: 557–560.

[14] Park Y-J, Baek JH, Lee S, Kim C, Rhee H, et al (2014) Whole genome and global gene expression analyses of the model mushroom *Flammulina velutipes* reveal a high capacity for lignocellulose degradation. PloS one 9: e93560. 24714189 10.1371/journal.pone.0093560 PMC3979922

[15] Maia LCD, Palmieri DA, Souza VQD, Kopp MM, Carvalho FIFD, et al (2008) SSR locator: Tool for simple sequence repeat discovery integrated with primer design and PCR simulation. Int J Plant Genomics 2008: 412696. 18670612 10.1155/2008/412696 PMC2486402

[16] Rozen S, Skaletsky H (2000) Primer3 on the WWW for general users and for biologist programmers. Methods Mol Biol 132: 365–386. 10547847 10.1385/1-59259-192-2:365

[17] Moreno AD, Ibarra D, Alvira P, Tomás-Pejó E, Ballesteros M (2014) A review of biological delignification and detoxification methods for lignocellulosic bioethanol production. Crit Rev Biotechnol Early online 1–13. 10.3109/07388551.2013.878896 24506661

[18] Chivukula M, Renganathan V (1995) Phenolic azo dye oxidation by laccase from *Pyricularia oryzae* . Appl Environ Microbiol 61: 4374–4377. 16535191 10.1128/aem.61.12.4374-4377.1995 PMC1388656

[19] Kudryavtseva O, Mazheika I, Solovchenko A, Kamzolkina O (2011) Genetic instability of the short-living ascomycetous fungus *Podospora anserina* induced by prolonged submerged cultivation. Microbiology 80: 784–796.

[20] Kudryavtseva O, Kamzolkina O, Mazheika I, Sellem C (2012) A mitochondrial respiratory mutant of *Podospora anserina* obtained by short-term submerged cultivation of senescent mycelium. Microbiology 81: 651–662. 23610920

[21] Park YJ (2013) s8: Mushroom Science: Complete genome and global gene expression analyses of the model mushroom *Flammulina velutipes* reveal a high capacity for lignin degradation, bioethanol production, and complex dynamics during mushroom formation. KSM newsletter 25: 25–25.

[22] Lohman TM (1993) Helicase-catalyzed DNA unwinding. J Biol Chem 268: 2269–2272. 8381400

[23] Rogers CW, Challen MP, Muthumeenakshi S, Sreenivasaprasad S, Whipps JM (2008) Disruption of the *Coniothyrium minitans* PIF1 DNA helicase gene impairs growth and capacity for sclerotial mycoparasitism. Microbiology 154: 1628–1636. 18524917 10.1099/mic.0.2008/017020-0

[24] Lange H, Sement FM, Gagliardi D (2011) MTR4, a putative RNA helicase and exosome co-factor, is required for proper rRNA biogenesis and development in *Arabidopsis thaliana* . The Plant Journal 68: 51–63. 21682783 10.1111/j.1365-313X.2011.04675.x

[25] Pérez G, Pangilinan J, Pisabarro AG, Ramírez L (2009) Telomere organization in the ligninolytic basidiomycete *Pleurotus ostreatus* . Appl Environ Microbiol 75: 1427–1436. 19114509 10.1128/AEM.01889-08 PMC2648151

[26] McEachern MJ, Iyer S (2001) Short telomeres in yeast are highly recombinogenic. Mol Cell 7: 695–704. 11336694 10.1016/s1097-2765(01)00215-5

[27] Nachman MW (2002) Variation in recombination rate across the genome: evidence and implications. Curr Opin Genet Dev 12: 657–663. 12433578 10.1016/s0959-437x(02)00358-1

[28] Stukenbrock EH, Croll D (2014) The evolving fungal genome. Fungal Biol Rev 28: 1–12.

[29] Xu J, Horgen PA, Anderson JB (1996) Somatic recombination in the cultivated mushroom *Agaricus bisporus* . Mycol Res 100: 188–192.

[30] Fierro F, Martín JF (1999) Molecular mechanisms of chromosomal rearrangement in fungi. Crit Rev Microbiol 25: 1–17. 10342097 10.1080/10408419991299185

