# Supplementary material for: Identification of Degenerate Nuclei and Development of a SCAR Marker for Flammulina velutipes
Source: PLoS One. 2014 Sep 15;9(9):e107207. doi: 10.1371/journal.pone.0107207 (PMC4164608; doi:10.1371/journal.pone.0107207)
Supplement: Figure S1 — Alignment of the sequences corresponding to the degenerate-specific region from the degenerate (D4), wild-type (W1) and KACC42780 strains. Gene prediction was conducted using FGENESH, and the protein function and domain analyses were conducted using the UniProt (http://www.uniprot.org/) program and the Protein BLAST program of NCBI. The exons of the putative helicase gene are shown in bold and the start and stop codons are underlined. * indicates the residues that are identical in the three sequences. ▾ and arrows indicate the sequence amplified using the SCAR marker primers that discriminated the degenerates. ∇ and arrows indicate the genomic sequences corresponding to the HELICc and HepA domains. The telomeric repeating units (TTAGGG) are in shaded boxes. (PDF) [file pone.0107207.s001.pdf]

```
Degenerate TACATCATTAATTACATCGGGAGTTGGTTGAATATATATCTAGCTCAACCAGATTTTCC-
Wild-type  TACATCATTAATTACATCGGGAGTTGGTTGAATATATATCTAGCTCAACCAGATTTTCC-
KACC42780  TACATCACCAATTACATCGGGAGTTGGTTGAATGTATATTCAGCCCAGCCTGACATTCCT
           *******  ************************ *****  *** ** ** **  ****

Degenerate TTCGAGTCCAATACTACAGTTTTACACCCTCCACGTAGTTAGCTGGTGTGTTCATTGAGG
Wild-type  TTCGAGTC----------------------------------------------------
KACC42780  TTTAAGTCCAATACTACAGCTTTGCACCCTCCCCATGGCTAGAAGGTGTGTTCATTGATC
           **  ****

Degenerate CAGAGCATACACATCAGGCCCGGATCAAAGGGCGAATTCTGCAGATATCCATCACACTGG
Wild-type  ------------------------------------------------------------
KACC42780  AGGAGGGACGATGTAGATGTG---------------------------------------

Degenerate CGGCCGCTCGAGCATGCATCTAGAGGGCCCAATTCTTGGCTAGAGCCTTAATACTTCATT
Wild-type  ------------------------------------------------------------
KACC42780  -----GCGTTGTATCTTTTCTGAGGGACGATTTACTTTGATAGAGCCTTAATACTTCATT

Degenerate ATGATTGTTATCCAGTTTTCTAGTATTTTGGGACCATTTCTTAATGTCAAGATCAATCGG
Wild-type  ------------------------------------------------------------
KACC42780  ATGTTTGTTATC------------------------------------------------

Degenerate TATATACACCAGCAAATACGCTCTAATGTGTTCGGTTGACTTTAATTAGGCCTTTCGCAG
Wild-type  ------------------------------------------------------------
KACC42780  ------------------------------------------------------------

Degenerate TCTCCGAACGTGTCTACAATGGTCTTCAATCGGGATCCTCGTCTGGCCGTAAGTTTCCGC
Wild-type  ------------------------------------------------------------
KACC42780  ------------------------------------------------------------

Degenerate CGCTATTCATGTCCGCCGTCTAATCCCGCCAGGGAGCGTTTATGAGCCGACCCGTAAGTT
Wild-type  ------------------------------------------------------------
KACC42780  ------------------------------------------------------------

Degenerate TTTGGCGGTAGACCCCGGTGCACCCCCGCAGCGACGTGGAACATTACGTTACGTCCCGGG
Wild-type  ------------------------------------------------------------
KACC42780  ------------------------------------------------------------

Degenerate CAGTATCTGCCGGGCCACCTCCATTTGACCGCCCGGATGCTTACCGGATTGTGGCCGTCG
Wild-type  ------------------------------------------------------------
KACC42780  ------------------------------------------------------------

Degenerate CACGTTTACCCCGTCGTGGGAAAGTCCTTCCGATGTGGCGCAACAGGTCGTGATCGTGGG
Wild-type  ------------------------------------------------------------
KACC42780  ------------------------------------------------------------

Degenerate AAAGGCAGGTGGCGGTGGGTATAAAAGCTGGATCCTCAATAACCAGGGTCTTTTGTCGCT
Wild-type  ------------------------------------------------------------
KACC42780  ------------------------------------------------------------
```

```
Degenerate TGAACTTGAACCACAACCTTCCTGTTCCACCCAGGCCAATTTCCAACACGCAGGGTCATT
Wild-type  ------------------------------------------------------------
KACC42780  ------------------------------------------------------------

Degenerate TTGTCGACCGTGAAGGTGCGTAGTCCGCATACGTGTGCCACATCACTTATCTGATCGGAT
Wild-type  ------------------------------------------------------------
KACC42780  ------------------------------------------------------------

Degenerate CACCCCTTTTAGCTTGCCACGTTTCCCTCAGTGTACCTTCCTCTATCTTCGCCTCGTGGA
Wild-type  ------------------------------------------------------------
KACC42780  ------------------------------------------------------------

Degenerate TCACTGCTAAAGATCCCCCAAGTACATCCGCCCGTTCATATCTCTTGCCCGTTGGCCCAG
Wild-type  ------------------------------------------------------------
KACC42780  ------------------------------------------------------------

Degenerate CTTCAGTCTGCGCCTCGGAACAACAGTGCGTCCTATTGGCACGTCTGTTTGTCATCACTG
Wild-type  ------------------------------------------------------------
KACC42780  ------------------------------------------------------------

Degenerate ATAACGTGTGGAGATCGTTCGTTCCGCTTTACACTCGTGTCGCTAAACGAGTCCCCGTCG
Wild-type  ------------------------------------------------------------
KACC42780  ------------------------------------------------------------

Degenerate ACCTCCCGGACCACTTGTGGTGAGTGATCTCCGGCCCAAAGGCTCCCACCGTATTGACTC
Wild-type  ------------------------------------------------------------
KACC42780  ------------------------------------------------------------

Degenerate TTGCTTTGATTTAGGCACACCGCTCCGAACAAACTAAGTGCGAACCGGTTACGTCGACAC
Wild-type  ------------------------------------------------------------
KACC42780  ------------------------------------------------------------

Degenerate CCGCCGTGGATAGACGCTCGCGGCTTGACCATCTCCGCTGTTGACTCCGCCCTCACAGAA
Wild-type  ------------------------------------------------------------
KACC42780  ------------------------------------------------------------

Degenerate GAATACCGCCGACTCTTCAAAAACTGATCTTGCCTAACGTGAGTAATGCTGCGTTCTTTC
Wild-type  ------------------------------------------------------------
KACC42780  ------------------------------------------------------------

Degenerate TTGGACGTGGTCGATATATGATACTCTAAATAGATGCATTGCGACGAAGGACTGGCGACG
Wild-type  ------------------------------------------------------------
KACC42780  ------------------------------------------------------------

Degenerate ACGATACGCTTCATTCACACCAATCTTGAGCTTCTTGACGACGTACAAGTCAACGTCGGT
Wild-type  ------------------------------------------------------------
KACC42780  ------------------------------------------------------------

Degenerate GACGAGCCCCAACTTATGCCGAGTCCGATGGCCACGATCATCGCAGAGAAGGGCATTCCT
Wild-type  ------------------------------------------------------------
KACC42780  ------------------------------------------------------------
```

Degenerate **GTCTTCCGGGATCCATCGGATGGGCCACCATGCCTCACGAAGGAAGAGAAGGCCGTCATA**
Wild-type --
KACC42780 --

Degenerate **ATCGCCAAGAGGAAAGGGGTCACCAATCAGGGGGCCGTCGTATACCTCGACTGGACGCCC**
Wild-type --
KACC42780 --

Degenerate **GACGCTTGGGAGTATGGCTGCCAGGTGGCGAGTGATCTCGTACAGCTGGGGTTGCGCCAG**
Wild-type --
KACC42780 --

Degenerate **GACATGACACAATACCGTTTCGACAATACGATGCCGGGCGGGA**AGCAGTTCTTGCATAAT
Wild-type --
KACC42780 --

Degenerate ATGAAGGTCCTACGGTACAAACATGCCTTCGAC**TCCGCCGTACGTTATTTCTTTTTTTCT**
Wild-type --
KACC42780 --

Degenerate **TCTTCCTCTTTTAAGAATGTATTTGTCCTAGATCATCCGCAGTCTGCGAGCACGCAAGTG**
Wild-type --
KACC42780 --

Degenerate **TACAATGGCGGATGTCTTTCATCGAGACATATCGAAAACATCAAAAATAGCGGCGAAAGC**
Wild-type --
KACC42780 --

Degenerate **CCCTGCCGCCCCGTCCTCGCCGGCGACCGGAAAGAAAGGAAAGAAGAAGCGGGGGGGAGA**
Wild-type --
KACC42780 --

Degenerate **AGGGGGAACAGATGATCACGCCATGCCCGGCTCCGCCATGTGGAATGCCGCCAGCAACGG**
Wild-type --
KACC42780 --

Degenerate **CATCATGAGCGACGTCTGCGGTCCCGCCATCCTCGTAGATGGTTATTTGGCGCCGGAGGA**
Wild-type --
KACC42780 --

Degenerate **CAGGACGTTCCTCACCGTCTTCCTCGATCGTTATCGCTCCAACTCCCTTCGGAACAAAGG**
Wild-type --
KACC42780 --

Degenerate **GCATTGTGAAAAGAAGCTCGCAGCGGCCAGAGCAAACGTCATTGCCCTTCTCGGAGCGGA**
Wild-type --
KACC42780 --

Degenerate **CTTCCTCACAT**CCGGCAAGGTTCCCGCCGACTTTACGCCACAACAAGCGCGAAAGGCCCT
Wild-type --
KACC42780 --

Degenerate TGGTGAACTGACCAAGTATTTTGTATGTTATTTGCTTCTTTCTCTTGTGATCCCTATCTT

Wild-type --

KACC42780 --

Degenerate ACTCCAGTGTACACAGAATTG**TCTAAAGTGGAACTTCCTGTCAAAGGCTGAACGTGC**GGC

Wild-type --

KACC42780 --

Degenerate TATGGCACTTCCCGTCGACGTAGACTCCCCCGGTGCTTTCGACCGTCTCGT**CAATCGTTG**

Wild-type --

KACC42780 --

Degenerate **GAACGCAATGTCCGAGCGTGCCCAAGCGCATGAGTGGTGTGCAACATCAATCGGCTGGAT**

Wild-type --

KACC42780 --

Degenerate **GGTTGAGATTTTGTCGCGGCGGTCCCGATCCCCGGTCAGAGA**GCCCGACATATATCCCGA

Wild-type --

KACC42780 --

Degenerate CGCTCTTATCGCATGTACGTTGTTATGATCATTTTG**CTTTTGACTCAAACTGAATGCATG**

Wild-type --

KACC42780 --

Degenerate **TCAGTTGAGAATGGCAAGTGTCTCGCACGAGCGATCAGCAAAAAGCAG**ATCAAGCGTCTG

Wild-type --

KACC42780 --

Degenerate GCCGAGACGCAGGAGCAAGACGTCATGCACTTCCAAC**GACATATGGAAGACTTCG**CCATG

Wild-type --

KACC42780 --

Degenerate GAGTTCCTCTCCGCCGACGAGACTTTTATTGATACCGAAGGCTTCCCTATAGGCGACCTG

Wild-type --

KACC42780 --

Degenerate GAAGTGGATCTCGCCGCTCTCAGAGACCAATGGAACAAGGAAAAGGATATGGGAATTGGC

Wild-type --

KACC42780 --

Degenerate GAGTTTGCCACTAAAGATGAGCAGGAGTTGCGGGAGTACTTGACAATCCGGGACG**GATGC**

Wild-type --

KACC42780 --

Degenerate **CCGGAGGCAATGAGGCGGTGGACGTGCGAGAATTACAAGCATATCCCGGTGGGGACCGCC**

Wild-type --

KACC42780 --

Degenerate **GAGAAGTTCGAAGGGGCCATCGAGTTCAAGCCGATGTGGCATCAGTTG**GCAGCGGTTGCC

Wild-type --

KACC42780 --

```
Degenerate GCTATATTCAAACGCCTGACTTTTACCGACCAGGCCGATGTCCACGACACGCTCCGGCAC
Wild-type  ------------------------------------------------------------
KACC42780  ------------------------------------------------------------

Degenerate GATCCATCCCACCCCGAGTCAGCCCCGCGTTCACTACGGGAAGGCTGGTCGCGTGTGCCG
Wild-type  ------------------------------------------------------------
KACC42780  ------------------------------------------------------------

Degenerate GGCACACTGATAGATGACGATGTCGGCTTGGGAAAGACTGTGACGACAGCGACGTGGATC
Wild-type  ------------------------------------------------------------
KACC42780  ------------------------------------------------------------

Degenerate GCGACCGTTATTCAGCTTTTAGAGGTCCAACGTGCCGCTGCAGCCACTTTGAGTCCGGAG
Wild-type  ------------------------------------------------------------
KACC42780  ------------------------------------------------------------

Degenerate GAACAGGTGAAGCATCGCCCGCGATGTCTCGGAGGTATGTATCCTGCTAGAAACTTTCTG
Wild-type  ------------------------------------------------------------
KACC42780  ------------------------------------------------------------

Degenerate GCGCAATGCTCATGCAATCTATACAGAAACTTTCGGCGGACTGAAAGAGATTCCTTCCGC
Wild-type  ------------------------------------------------------------
KACC42780  ------------------------------------------------------------

Degenerate CCCGCACATCATCGTTGTCCCAAGCGGTCTAATCCCTCAGTGGGTCGGCGAGTTGAAGCG
Wild-type  ------------------------------------------------------------
KACC42780  ------------------------------------------------------------

Degenerate ATTTTTCAAGCAAGGAGCGGTCCAAATTGTGGTTGTCCGGACCGGCGGGGAGTTATGGAA
Wild-type  ------------------------------------------------------------
KACC42780  ------------------------------------------------------------

Degenerate GGAAGATGTTAAGACGCTGTTCGCTGATGGACCGGTCCCGCTTCATCGCCGCATTATGCT
Wild-type  ------------------------------------------------------------
KACC42780  ------------------------------------------------------------

Degenerate GGTGCAGGTCAGCGTGAGTGCTATGTCATAAGCTTTCCGGTATATCCCTAACATCAGTGT
Wild-type  ------------------------------------------------------------
KACC42780  ------------------------------------------------------------

Degenerate TGTAGACATTTCAGGGCAAGATGTCCAAGGGCGCATACAAGAAGGACGGCGATGCTCGTA
Wild-type  ------------------------------------------------------------
KACC42780  ------------------------------------------------------------

Degenerate TTAGCCTTATCTTCCCCAACACCGTTGAGTTCATCCACACCTACAGCTTCCTCAGTGCCT
Wild-type  ------------------------------------------------------------
KACC42780  ------------------------------------------------------------

Degenerate GGATTGACGAGGCTCACGGTGCGAGAACGTGCAACATGTTATGGCATGCTATCGAGGCAC
Wild-type  ------------------------------------------------------------
KACC42780  ------------------------------------------------------------
```

```
Degenerate TCTTCCATGTCACTCTGGTCAAAGTCCTGTTGACCGCCACACCATTACTACAAGCTGCCG
Wild-type  ------------------------------------------------------------
KACC42780  ------------------------------------------------------------

Degenerate CCGTAAGTTCTCATTCATACGCATTATGCCTACTAAAATCATATATAGGACTTGTATTAT
Wild-type  ------------------------------------------------------------
KACC42780  ------------------------------------------------------------

Degenerate CTCGCAGTTTTGCTGAGGCCGGCTAAGCTCACTGAAGCAAAACGACGAGAAATCAGAGGG
Wild-type  ------------------------------------------------------------
KACC42780  ------------------------------------------------------------

Degenerate CTTGTCATGGAAGGGAAGCGAGCAAAGGCAAAGAGCGCTCTCCTTTCAGGGACTCATCAT
Wild-type  ------------------------------------------------------------
KACC42780  ------------------------------------------------------------

Degenerate TTCGCTTCCAAATCGGAGAAGGAGATGCGGGATGCCGCCGCTGCATCACTGGTTCACAAG
Wild-type  ------------------------------------------------------------
KACC42780  ------------------------------------------------------------

Degenerate ATGGGCATGCGTGTCGTCATTGCAACGCAGGCGGTTCTCATGGAGTGCTGCATTCGGCGA
Wild-type  ------------------------------------------------------------
KACC42780  ------------------------------------------------------------

Degenerate ACCGGAAAGTCGCTCCGGTGGGATGGCACACCGATCAACGCGGATATCCCGCAAAACACG
Wild-type  ------------------------------------------------------------
KACC42780  ------------------------------------------------------------

Degenerate ATGACTTACTGCATGGTCGACGTCAGTGACGAGGAGGCGGAGGCAGCGGGGAAGGCGGTG
Wild-type  ------------------------------------------------------------
KACC42780  ------------------------------------------------------------
                                        ▼→
Degenerate GAGGAGGAAATCGCCAATGGTAAAGGATTTGAAGCGGACAAGATGGGGGTGCGTCGCTAA
Wild-type  ------------------------------------------------------------
KACC42780  ------------------------------------------------------------

Degenerate CATTTTCCGGCGCTGCTTTACTAACTATCGTGCAGAAATTCTACCTCGGACCCAAGCTTA
Wild-type  ------------------------------------------------------------
KACC42780  ------------------------------------------------------------

Degenerate TCCTTGGTTGCAAGGACATCGACAAGATATCACCGACCGAACCACTGACTTTGGAGCAAT
Wild-type  ------------------------------------------------------------
KACC42780  ------------------------------------------------------------

Degenerate GCAAAGAACTCTATATGTCCAAAGCCAAGGTTGCCGTCGAGCTCTCGAAAGCTATCGTCC
Wild-type  ------------------------------------------------------------
KACC42780  ------------------------------------------------------------

Degenerate ATCGCGGTCCGGCCGCCGTTGTTCCCCAGGAGCTACACGGAACGGACGAATCCATTTCCC
Wild-type  ------------------------------------------------------------
KACC42780  ------------------------------------------------------------
```

Degenerate **CCCAGTTCCCTAGCCAGGACCGCTTCGGCATCCCTTGCGTTTACAAGGACTTTCATCCAG**
Wild-type --
KACC42780 --

▽→
Degenerate **AGCCGCTCAAGCCGGGAGATGCAAT**TCCCGGTGAAAAGGTCATAATCCACACGCTCTTTG
Wild-type --
KACC42780 --

Degenerate CCATGTTCCATGAG**CTGCTCACGCAGGTACGTCTTGTGCTTCCCGCCCTCCATCTGCCTG**
Wild-type --
KACC42780 --

Degenerate **ACCATCTCCGGCACAGATGTACGCAGTGAACGGCATGAAGGCGGTATCCATATCGGGCAA**
Wild-type --
KACC42780 --

Degenerate **GGTCCACGGGGACGGGCGTGATAAAGTCATCGAGGAATTCAAGACGGACCCTACCATCCA**
Wild-type --
KACC42780 --

Degenerate **AGTTCTCA**TCATGAGCAATGTCGGTACGGTGGGGCTGAACTTGACCGTCGCCGCGTCA**CT**
Wild-type --
KACC42780 --

Degenerate **CATTCTATTTGTGCGTATCTTCCGCGTCTGAAAGGTTCCGGCATTTATTCACTCAACAGG**
Wild-type --
KACC42780 --

Degenerate **ACGTCAATTGGTCGGGTGTAATGGCCCACCAAATATATGGTCGAATCCATCGTCGTGGAC**
Wild-type --
KACC42780 --

Degenerate **AAACACGCGAGACCTTTATCATACAACTGGTTTGCGCGAGGACGGTTGAAGTCTTGCTAG**
Wild-type --
KACC42780 --

Degenerate **TGGCCAACGGTCTGTCTAAACACACACTTCTTTGCAACTTCCTTGAGGCAGAGCGCAATG**
Wild-type --
KACC42780 --

Degenerate **AAGGTATGTCAGTTTATGTTGATGCACGTCAATACTAACAATAGTGCAGAGCTGTGGAAT**
Wild-type --
KACC42780 --

Degenerate **CTAATCCAAAACCGGGACAGTTGTGACGATAACCTGCCCGCTGTCGACG**CACTAGCCGGA
Wild-type --
KACC42780 --

Degenerate TTCGACGACGACGCCCGTGCGCAAGCGAAGGCAGCC**AAAGTCAAGGTCCGTGCCACTGCC**
Wild-type --
KACC42780 --

Degenerate **GGTCCGTCGAAAGCCAAGAAGCGGAAAGTCACAAGTGTCGAGACCGTTGGGGACGACGAT**
Wild-type --
KACC42780 --

Degenerate **GCCGTTACTTCGAAGGCGGTGCGCCCCAAGAAGGGAAAGGCTCCCGTCGGCGAGATCGTG**
Wild-type --
KACC42780 --

Degenerate **GTTGACAACCCCGCCGCTGCTCCATCTACTGGACCTTCCAAAGGAAAGGGCAAGGGCAAA**
Wild-type --
KACC42780 --

Degenerate **GCCTCCATACCCATAGACGTCAGTGACGACTCGGAGGAGGAGGAGGAAGATGATGCACTG**
Wild-type --
KACC42780 --

Degenerate **CCTCCCGCCAAGAAGAAGAAGGGGGGGAAAGAGGTGGCGCTGCCTACGGCGGGGGTCGGA**
Wild-type --
KACC42780 --

Degenerate **GGGTCGAAGAAACGGAAGACTCTAGCTGGGAGCAACGCCTCACAGCCTCCTTCGCCTGTC**
Wild-type --
KACC42780 --

Degenerate **AAACCTCCGGCGAAAAAGAGCAAGACTGACATTCCG**GCATCCCCGTCCACCGCTCCTCCA
Wild-type --
KACC42780 --

Degenerate TCTCCGATGCACGTTGACGCCC**CGCACGAAGTGACTGGATCTCCGACGCCGCCATCTCCC**
Wild-type --
KACC42780 --

Degenerate **TCTCCCGTCAAACCTCCGGCGAAAAAGAGCGAGACCGACATTCCGGCAACTCCGTCCACC**
Wild-type --
KACC42780 --

Degenerate **GCTCCTCCATCTTCCGTGCACGTTGCCGCCCCGCCCAGATTGACTCGATCTCCGCCGCCG**
Wild-type --
KACC42780 --

Degenerate **CCGGGCCGGAAACGTGCCGCTACTCCCCCGTCTCCGTCCCCATTTTCCGCAAAGAGCAAA**
Wild-type --
KACC42780 --

Degenerate **GGCAAGCAACGCGCCACTA**CTCCGCCTCCGCCATCTCTGAAACGGAAGAAGCAACAACCT
Wild-type --
KACC42780 --

←▽
Degenerate CCGCCTCCGCC**TCTCCCTCCTCCGTCGCCTTCTCTCCCAGCAGTGGATGATGACGACTTG**
Wild-type --
KACC42780 --

```
Degenerate TACACCGGGGTTTTCGATGAGTTCATCGGCGATTTCGAGGAGGATGACCGTCCGCGAGAG
Wild-type  ------------------------------------------------------------
KACC42780  ------------------------------------------------------------

Degenerate GAGAAGAAATCCTTGAACGTAGGCCCTACGTTGTATAAAAAGCCACTGCGGGACGGCGGC
Wild-type  ------------------------------------------------------------
KACC42780  ------------------------------------------------------------

Degenerate CGTTGGTACATTGCCGATAAAGTGGCCGTTCCTGACGACTTTCCCTTCAGCGGCCTTTCG
Wild-type  ------------------------------------------------------------
KACC42780  ------------------------------------------------------------

Degenerate GTGGCGGACTGGAACACTTACTATAAACAACCTCAGATTTTCCCAGACTCACCGACGCAT
Wild-type  ------------------------------------------------------------
KACC42780  ------------------------------------------------------------

Degenerate ACGGCGGTGTGGGAACAATGGAACAACAAACACTATGCTGCCAATGTCGGGGATTGGGAT
Wild-type  ------------------------------------------------------------
KACC42780  ------------------------------------------------------------
                                                           ←▼
Degenerate CTCGACTCGGGTCTGCCCCCTCCAGTTTCCCCGTCGACTTCATTGTCCGCTCTTAGCTCG
Wild-type  ------------------------------------------------------------
KACC42780  ------------------------------------------------------------

Degenerate CTTCCTGACCACTTCAAGTCTACGCAGCCTTCCGAGGACGACAGTTCCGACAACGAAGAT
Wild-type  ------------------------------------------------------------
KACC42780  ------------------------------------------------------------

Degenerate GACAGCCCTGTTCATGCCCCAAAGACGAAGCCAGCAAAGGTATGTGTAGCATTTCACAAT
Wild-type  ------------------------------------------------------------
KACC42780  ------------------------------------------------------------

Degenerate ATCTGATCTAACCTCGTAATGAATAGCCTTCCCGGTGGAATATCAACCCCGAAGTCCGGG
Wild-type  ------------------------------------------------------------
KACC42780  ------------------------------------------------------------

Degenerate AAACCAAATCTAAGGTTGTTGCGAAGCCGCCTGTTATCCCCCCGCCCATGTCCGTTCGGA
Wild-type  ------------------------------------------------------------
KACC42780  ------------------------------------------------------------

Degenerate AGCCGGGAGTGTCCGCGAAGAAGAAGTTGGTGAAAGGAAAGCCATCGGGATCGAAGAGAC
Wild-type  ------------------------------------------------------------
KACC42780  ------------------------------------------------------------

Degenerate CTACATGAGATACATCGCCACATAGAATATTCCTTCTTGTATTTGTAATCAAGCAGCAGT
Wild-type  ------------------------------------------------------------
KACC42780  ------------------------------------------------------------

Degenerate TGAATTTTCACCGGCGAATTTTCTTCCCAGTACGATTGAAGCATGTTCGTGATACATATT
Wild-type  ------------------------------------------------------------
KACC42780  ------------------------------------------------------------
```

Degenerate **TACTTCGCGGACTATGTATCCCGTGCCAGAAATATTCTGGCAAGCACAGGTAAAGGTGAA**
Wild-type --
KACC42780 --

Degenerate **CTTATATATGGTCTGCATTTCACCGAATGGCACAGGTCTTACACCGCGCCAGAAATATTC**
Wild-type --
KACC42780 --

Degenerate **TGGCAAATGCAACCACAC**CAACCGCCGGGCAACTTTAACTCGGTTATGGTATACATGTAT
Wild-type --
KACC42780 --

Degenerate ACCATAACCGA**GTTAAAGAGTCCTGATACATGGATCATTTAGACGGTTGAAAATACTGCC**
Wild-type --
KACC42780 --

Degenerate **TGGGGCGATACTTTACGGACTACTTATCCCGCGCCAGAAATATTCTGGCGAACACAACCA**
Wild-type --
KACC42780 --

Degenerate **CACCAACCGCCGGGCAACTCTAACTCAGATATGGTGTATCAAGAGTTCCGATAACACTCA**
Wild-type --
KACC42780 --

Degenerate **AAGTGATTCAGACACTGTAAAACATAGCCTGCCTTCGTGGACTATGTGTTCCTTATCACA**
Wild-type --
KACC42780 --

Degenerate **TTCCGGTG**AGTACAGGCGAACTGCCATGCTGTTTACGTTTCTCATCTTCGCATAACTAAC
Wild-type --
KACC42780 --

Degenerate CA**TTTGAAATTCAAACTATTTGAAATACCAGTGACTCTGATGACAAAGCAAGCTCTGTGG**
Wild-type --
KACC42780 --

Degenerate **TATATATTCCAGCACATCCCCCGTTCTCTCCTTCCTTCTCCCCTCCATCATGGACTACGA**
Wild-type --
KACC42780 --

Degenerate **TGGTGACGAGGATCTCGATCGTGACCCCGACTTCAACTACAATCCTGACATTGAAGATGT**
Wild-type --
KACC42780 --

Degenerate **CACCCTCCCTGAGATCATAACACTATTCGACGACGCTCGTCTGCTTGCTCTTGGTGCGGC**
Wild-type --
KACC42780 --

Degenerate **GCTCGATCCCGGCGCGGATTGGGCCGGCATGGACAAGGACGCCGTTCAGAAGTACATTGC**
Wild-type --
KACC42780 --

```
Degenerate TTTAACTCTGTGCGGCACCAAGGAAGGCGACGACCCCGTGCGGTACAATCTCGACTTTGA
Wild-type  ------------------------------------------------------------
KACC42780  ------------------------------------------------------------

Degenerate CAACGCCTACCAGGATGTTTACGACCACCCCGACGCCAAAGTCATGAAAGACATTGATTC
Wild-type  ------------------------------------------------------------
KACC42780  ------------------------------------------------------------

Degenerate TGCCCTCGTCTTCGGGTCCAAGTTTTTCTACACCGACAACTATGATATAATGACCACGTT
Wild-type  ------------------------------------------------------------
KACC42780  ------------------------------------------------------------

Degenerate CGATGCCAAGTTCACTCTCGGCGGTCATCTTCATTGCAAGGTTCCGTTCACGGTAAGTGA
Wild-type  ------------------------------------------------------------
KACC42780  ------------------------------------------------------------

Degenerate CCTGTCACTTGTAATGGCAAGTTTACTCACATTGGCGTAAGAATCCACTCAGCCCCGACG
Wild-type  ------------------------------------------------------------
KACC42780  ------------------------------------------------------------

Degenerate AGAATGTTTACCGTGATCCCGGCGATGATACAGCTGTCTTCTGGGGCAAATGTGGAATTG
Wild-type  ------------------------------------------------------------
KACC42780  ------------------------------------------------------------

Degenerate CCGGTCGTGATCGCATTCATCTCATGCTTCCTGACGCCGGCAATGTTCCTCTTATGGAAA
Wild-type  ------------------------------------------------------------
KACC42780  ------------------------------------------------------------

Degenerate TTCACAGCGCGATCTATGCTGCGAATCTCACCGCCGCCAAGGAAATCCTTCCGGACGTCG
Wild-type  ------------------------------------------------------------
KACC42780  ------------------------------------------------------------

Degenerate TCAAGCAGTGGTCTCCGGATTATCAAACGGAACTAAGCCGGACTGGGTACTGGCGCCCTA
Wild-type  ------------------------------------------------------------
KACC42780  ------------------------------------------------------------

Degenerate ATCCAACGCGTCGCACTCAGTCTACTCATCCCATCCGAGGCGGCGACATAGACGCGGTTA
Wild-type  ------------------------------------------------------------
KACC42780  ------------------------------------------------------------

Degenerate ACGATATACAAACCGACGGTGCTTTGTTCGGTACACGCGTCATGGAGATTATCCGCACAT
Wild-type  ------------------------------------------------------------
KACC42780  ------------------------------------------------------------

Degenerate ATGACTGGGGGAAGCACGCCTACTGGTTTCTTCAAATCCGCGGTGCTAAAGACCTGACAC
Wild-type  ------------------------------------------------------------
KACC42780  ------------------------------------------------------------

Degenerate GCCATACCGGCGGCTCCGCCAATACGGAAGAGTGGATCCAAACCCTCCTTACCTACGTTC
Wild-type  ------------------------------------------------------------
KACC42780  ------------------------------------------------------------
```

```
Degenerate ATGAACCCTTCTGCGCAATTTACCTCGATATGGCAGTGGAACTTCATCTTCCCGCTGGAT
Wild-type  ------------------------------------------------------------
KACC42780  ------------------------------------------------------------

Degenerate ACTCCTCTCTTATCCGTCGCGGGGATGAGACGGTCGAGGGTTTAGCGGAGACCCTTTTCA
Wild-type  ------------------------------------------------------------
KACC42780  ------------------------------------------------------------

Degenerate ATGTCCCGCCAGAGGTGTTCGACACAGTCGACGTAGAGCCGGACGTGTGGGCCGGAGTAC
Wild-type  ------------------------------------------------------------
KACC42780  ------------------------------------------------------------

Degenerate AGCAGTCGGCGGGCTTTCGCGCCAACTTCACCGCAAACCCGGTCACCGTTTACCGCATCT
Wild-type  ------------------------------------------------------------
KACC42780  ------------------------------------------------------------

Degenerate CATACATCCAGGCGTATACCACGGACAAGTTCCAGACATACAACGTCTCCTCCTTCTCAT
Wild-type  ------------------------------------------------------------
KACC42780  ------------------------------------------------------------

Degenerate CCAAGTCCATGAAGCTGTATCCACAAACCGCGTTCAAAATGAAAACAGCGGACGACATTC
Wild-type  ------------------------------------------------------------
KACC42780  ------------------------------------------------------------

Degenerate CGGTGTCGTATCCTCTTGTCTATGACGCGCTCCTCACCAACCTGGGAGCAAAGACGCCTG
Wild-type  ------------------------------------------------------------
KACC42780  ------------------------------------------------------------

Degenerate GAGCTGTCCGTGTCGAAGTACGTGTGCCGCGCAGTCAAGCCAATGCTGTCTTTGCTCTTG
Wild-type  ------------------------------------------------------------
KACC42780  ------------------------------------------------------------

Degenerate CCATGGAACCGCGCGACTTTCAAGGGTACACGCATATAGTGAAGAACGAGATGTTATGGT
Wild-type  ------------------------------------------------------------
KACC42780  ------------------------------------------------------------

Degenerate ATGTCATGTGCCAGAATCTTTCTGGCATATATGCTTACATGGATCGCAAACAGGGCATGG
Wild-type  ------------------------------------------------------------
KACC42780  ------------------------------------------------------------

Degenerate CGTCTACACAAAATCTGTGCTTGCTTGTTGTTACTCCGCTACGCTGCCTCTTGCCGTCGC
Wild-type  ------------------------------------------------------------
KACC42780  ------------------------------------------------------------

Degenerate GACACCCGCGCCCATAAGGCCACCCTTCTACTTGTTGCCGCAACTACATTTGTCTTCAAC
Wild-type  ------------------------------------------------------------
KACC42780  ------------------------------------------------------------

Degenerate TCCATGCATTCACGGCCGATGGAGATGGGATGGGACCGGCAGGTGGGAGCTCTCATTCTC
Wild-type  ------------------------------------------------------------
KACC42780  ------------------------------------------------------------
```

```
Degenerate CCTATCGGCACCGTCGCACAGAGCTCGGAGAACAAGGATCGCATGATTTGGGTTAGGGGG
Wild-type  ------------------------------------------------------------
KACC42780  ------------------------------------------------------------

Degenerate GGTTAGGGTTAGGGGGGTTAGGGTTAGGGG
Wild-type  ------------------------------
KACC42780  ------------------------------
```

Supplemental Fig. S2. Alignment of the sequences corresponding degenerate specific region from degenerate (D4), wild type (W1) and KACC42780. Gene was predicted by FGENESH, and function and domain were estimated by UniProt (http://www.uniprot.org/) and Protein BLAST in NCBI. Exons of the putative helicase gene are shown in bold and start and stop codons are underlined. * indicates the identical residues in three sequences. ▼ and arrows indicate the sequence amplified by the SCAR marker discriminating degenerates. ▽ and arrows indicate the genomic sequence corresponding to HELICc and HepA domains. Telomeric repeating units (TTAGGG) are in shade box.
