# Supplementary figures and images for: Identification of Degenerate Nuclei and Development of a SCAR Marker for Flammulina velutipes
Source: PLoS One. 2014 Sep 15;9(9):e107207. doi: 10.1371/journal.pone.0107207 (PMC4164608; doi:10.1371/journal.pone.0107207)

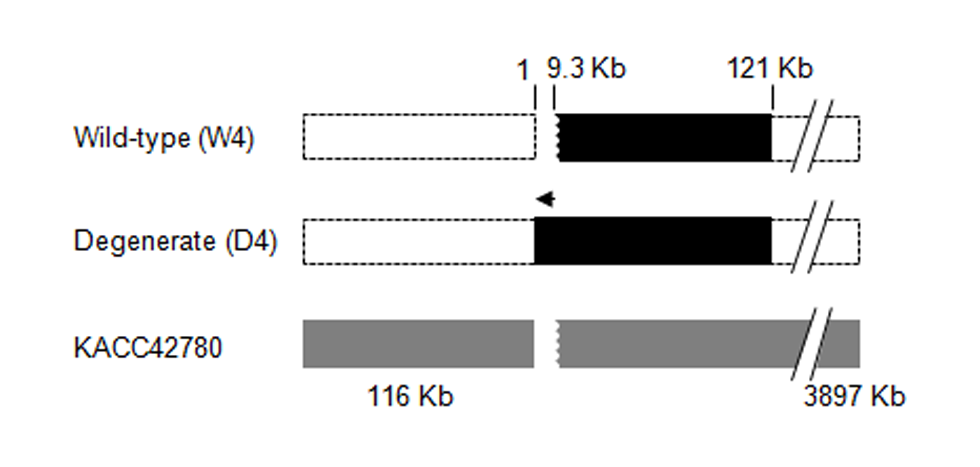

Supplement: Figure S2 — Deduced map of the genomic region corresponding to the degenerate-specific sequence of the wild-type (W1), degenerate (D4) and KACC42780 strains. Unknown but deduced sequences are shown in boxes indicated with a broken line. The arrow indicates the direction of transcription of the putative helicase gene. The black boxes in the W1 and D4 sequences indicate high similarity (99%), and the gray boxes indicate low similarity (30 to 70%). (TIF) [file pone.0107207.s002.tif]
