# Supplementary material for: Identification of Degenerate Nuclei and Development of a SCAR Marker for Flammulina velutipes
Source: PLoS One. 2014 Sep 15;9(9):e107207. doi: 10.1371/journal.pone.0107207 (PMC4164608; doi:10.1371/journal.pone.0107207)
Supplement: Table S1 — List of primers used in this study. (DOCX) [file pone.0107207.s003.docx]

**Supporting Information**

**Table S1. List of primers used in this study.**

| Primer name | Sequence (5′ to 3′) |
| --- | --- |
| FEDE_F | TGAGCGGACAAGATGGGGGTGC |
| FEDE _R | TGAGCGGACAATGAAGTCGACG |
| 1-1000_F | CGGTGCCGATAGGGAGAATG |
| 1-1000_R | GCAAACCCGGTCACCGTTTA |
| 1000-2000_F | ATTCTCGTCGGGGCTGAGTG |
| 1000-2000_R | CAGCACATCCCCCGTTCTCT |
| 2000-3000_F | TGTGCCATTCGGTGAAATGC |
| 2000-3000_R | CCCCGTCGACTTCATTGTCC |
| 3000-4000_F | GGACGGTCATCCTCCTCGAA |
| 3000-4000_R | CAACGCCTCACAGCCTCCTT |
| 4000-5000_F | ACTTTGGCTGCCTTCGCTTG |
| 4000-5000_R | CTGACCATCTCCGGCACAGA |
| 5000-6000_F | TATCCGCGTTGATCGGTGTG |
| 5000-6000_R | CGGCGATGCTCGTATTAGCC |
| 6000-7000_F | TCCGTCCCGGATTGTCAAGT |
| 6000-7000_R | GGTGCTTTCGACCGTCTCGT |
| 7000-8000_F | ACCTTGCCGGATGTGAGGAA |
| 7000-8000_R | GTTGCGCCAGGACATGACAC |
| 8000-9000_F | GTCTATCCACGGCGGGTGTC |
| 8000-9000_R | GGTCGTGATCGTGGGAAAGG |
| 9000-10000_F | TGATCCGGGCCTGATGTGTA |
| 9000-10000_R | CTGTCTGCCACGGATCCTCA |
| 9244-10004_F | TGATCCGGGCCTGATGTGTA |
| 9244-10004_R | TGGTCATGAGTACACAGTTGTGCAG |
| 9500-10500_F | CCAATTCCTTCGGGACAACAAA |
| 9500-10500_R | GTGGTCATGAGTACACAGTTGTGCA |
| 11000_F | CCTTCAAAGACATGACCCTACT |
| 11000_R | GGCTAGGGTCCCATAATAATGTA |
| 12000_F | CTTTTGTTCGTGTCTGCACCAA |
| 12000_R | GCTTCTGTCCATTGTTCAGAACTA |
| 13000_F | GCTAGCAGTACCAACGGAAG |
| 13000_R | GCCACCACTGTGAGGCCC |
| 14000_F | TAACACGTCGACGGCGTCC |
| 14000_R | GGCTCCGCCTGCATGCTG |
| 15000_F | AGAGAGAAGACAGCGGGGAG |
| 15000_R | GGAAGCGTGCCACCGCAT |
| 16000_F | GCCACTCAGTACGAGTGGC |
| 16000_R | GTAATCAAAGTGGATGCGGTC |
| 17000_F | TCATCCCCTTGGGCAGCG |
| 17000_R | CCTTGTCCAGCATTGGGAGC |
| 18000_F | GTTGGGAATGGTAGGCATGA |
| 18000_R | GAGGAGACCCTGCCAGAC |
| 19000_F | ACCGAAGAATGACTAGCGTTAC |
| 19000_R | GTATTCACATTTATTGATTATAAACAGAC |
| 20000-21000_F | GTGGGTGAAAGGGAAGCAAG |
| 20000-21000_R | TCTGCTCAAGGCCAGTGAAA |
| 30000-31000_F | CCACCATGACCCCGTATCTT |
| 30000-31000_R | TACCACCGGCACATTGTCTC |
| 40000-41000_F | AGGGGCCCTACAAGAAGCTC |
| 40000-41000_R | CAGAGAAAGTGCGCATCAGG |
| 50000-51000_F | CCTTGTCATCCGACAAGCAA |
| 50000-51000_R | ATTGCAGCAGGCAGAGATGA |
| 60000-61000_F | ACGGGTCGATGTCCTGCATT |
| 60000-61000_R | GCCAAATGGTGCGCGATAAT |
| 120005-121005_F | AGCCCCTTGCAACTGTCGAG |
| 120005-121005_R | GGCCTTGGTTTCTGGCTTGA |
